# Supplementary material for: Mechanical confinement governs phenotypic plasticity in melanoma
Source: Nature. 2025 Aug 27;647(8089):517–27. doi: 10.1038/s41586-025-09445-6 (PMC12611772; doi:10.1038/s41586-025-09445-6)

nature portfolio

https://doi.org/10.1038/s41586-025-09445-6

Supplementary information

Mechanical confinement governs phenotypic plasticity in melanoma

In the format provided by the authors and unedited

Figure 5f: HMGB2

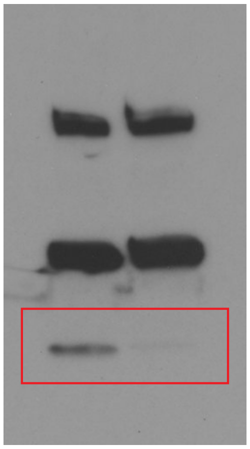

25 kDa

Figure 5f: tubulin

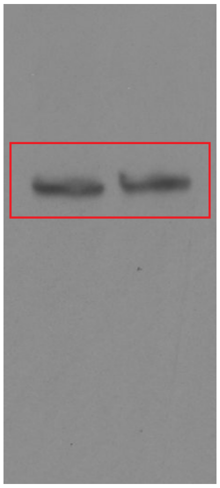

50 kDa

Figure 5h: HMGB2

kDa

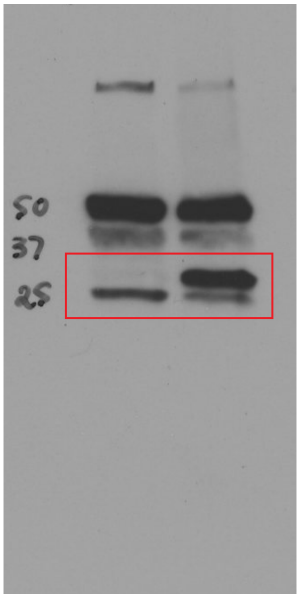

Figure 5h: β-actin

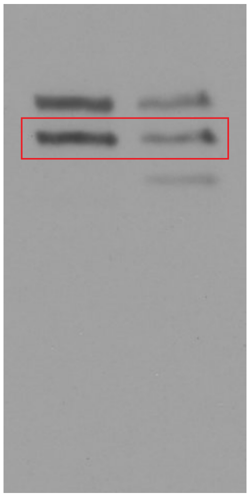

42 kDa

Extended Data Figure 8b: HMGB2

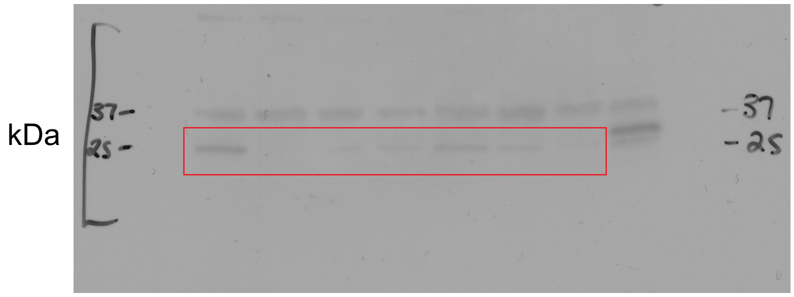

Extended Data Figure 8b: tubulin

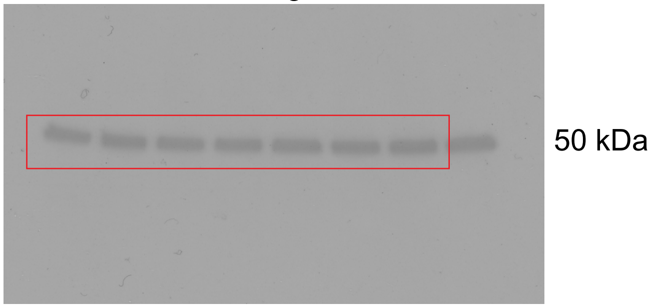

Extended Data Figure 11f: HMGB2

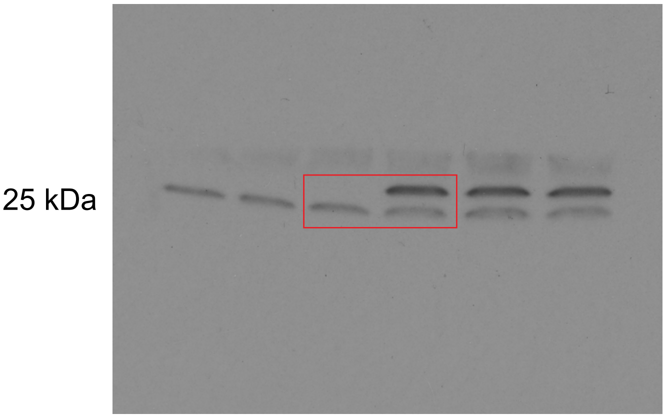

Extended Data Figure 11f: tubulin

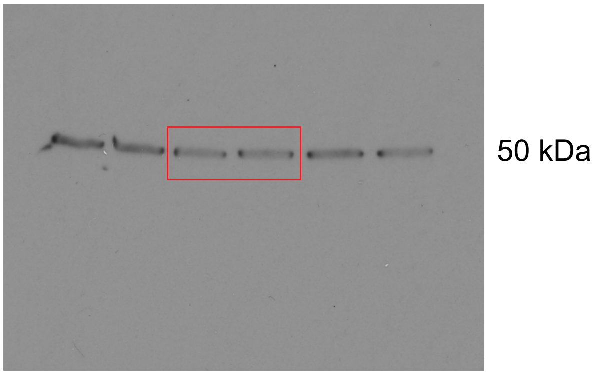

Extended Data Figure 12a:

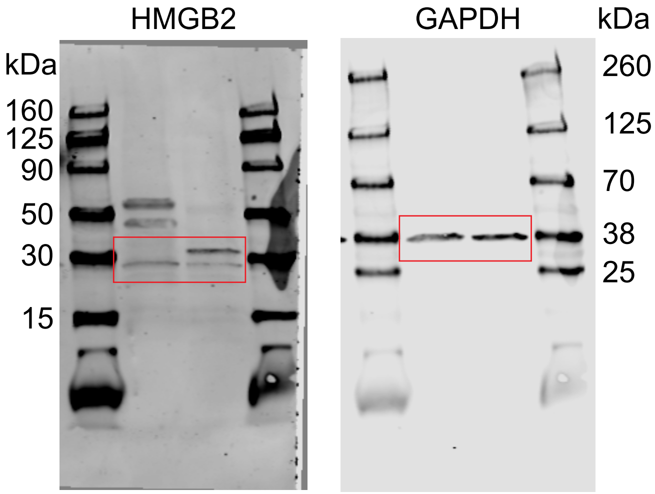

Extended Data Figure 12e:

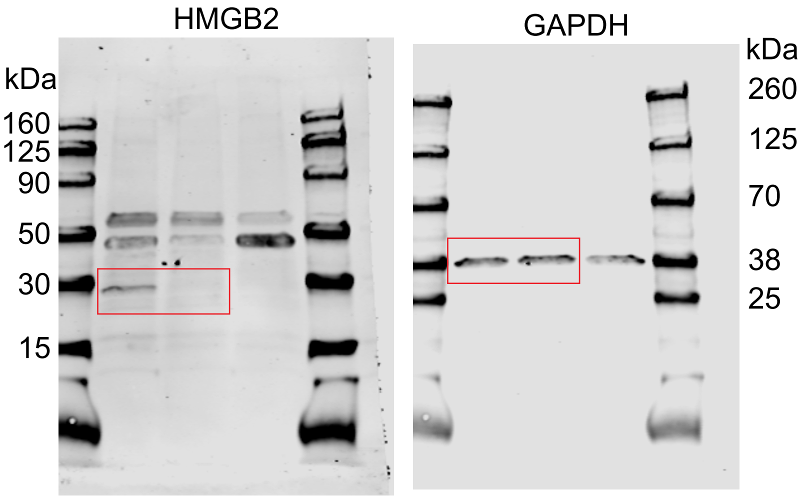

Supplement: Supplementary file 1 — Full scans of western blots. Uncropped gels for Fig. 5f,h and Extended Data Figs. 8b, 11f and 12a,e. [file 41586_2025_9445_MOESM1_ESM.pdf]
