# Supplementary material for: Mechanical confinement governs phenotypic plasticity in melanoma
Source: Nature. 2025 Aug 27;647(8089):517–27. doi: 10.1038/s41586-025-09445-6 (PMC12611772; doi:10.1038/s41586-025-09445-6)
Supplement: Supplementary file 2 — Reporting Summary [file 41586_2025_9445_MOESM2_ESM.pdf]

nature portfolio

Corresponding author(s): Miranda V. Hunter, Richard M. White

Last updated by author(s): 6/11/2025

Reporting Summary

Nature Portfolio wishes to improve the reproducibility of the work that we publish. This form provides structure for consistency and transparency in reporting. For further information on Nature Portfolio policies, see our Editorial Policies and the Editorial Policy Checklist.

Statistics

For all statistical analyses, confirm that the following items are present in the figure legend, table legend, main text, or Methods section.

n/a	Confirmed	
☐	☒	The exact sample size (*n*) for each experimental group/condition, given as a discrete number and unit of measurement
☐	☒	A statement on whether measurements were taken from distinct samples or whether the same sample was measured repeatedly
☐	☒	The statistical test(s) used AND whether they are one- or two-sided<br>*Only common tests should be described solely by name; describe more complex techniques in the Methods section.*
☒	☐	A description of all covariates tested
☐	☒	A description of any assumptions or corrections, such as tests of normality and adjustment for multiple comparisons
☐	☒	A full description of the statistical parameters including central tendency (e.g. means) or other basic estimates (e.g. regression coefficient) AND variation (e.g. standard deviation) or associated estimates of uncertainty (e.g. confidence intervals)
☐	☒	For null hypothesis testing, the test statistic (e.g. *F*, *t*, *r*) with confidence intervals, effect sizes, degrees of freedom and *P* value noted<br>*Give P values as exact values whenever suitable.*
☒	☐	For Bayesian analysis, information on the choice of priors and Markov chain Monte Carlo settings
☒	☐	For hierarchical and complex designs, identification of the appropriate level for tests and full reporting of outcomes
☐	☒	Estimates of effect sizes (e.g. Cohen's *d*, Pearson's *r*), indicating how they were calculated

Our web collection on statistics for biologists contains articles on many of the points above.

Software and code

Policy information about availability of computer code

Data collection	Zen Black v2.3 SP1 software was used to acquire imaging data.
Data analysis	Custom R and MATLAB code used for analysis. R versions: 4.3.1; MATLAB versions: R2021b and R2023b. Other software/packages used for bioinformatics analyses: Seurat v4.4.0 and 5.0.1; HOMER v4.5 and v4.11, fgsea v1.26, TrimGalore v0.4.5 and 0.6.7, FastQC v0.11.5 and v0.12.1, cutadapt v1.15 and v4.0, bowtie2 v2.3.4.1 and v2.3.5.1, Picard v2.16, deepTools v3.3.0 and v3.5.1, clusterProfiler v4.10.0, DESeq2 v1.42, featureCounts v1.6.1. Additional software/plugins used for image analysis: CellProfiler v4.2.5, TrackMate v7.11.1, Fiji v2.14, Spectronaut v18.5. All code used for analysis and plotting is available at github.org/mvhunter1/Hunter_2024.

For manuscripts utilizing custom algorithms or software that are central to the research but not yet described in published literature, software must be made available to editors and reviewers. We strongly encourage code deposition in a community repository (e.g. GitHub). See the Nature Portfolio guidelines for submitting code & software for further information.

Data

Policy information about availability of data

All manuscripts must include a data availability statement. This statement should provide the following information, where applicable:

- Accession codes, unique identifiers, or web links for publicly available datasets
- A description of any restrictions on data availability
- For clinical datasets or third party data, please ensure that the statement adheres to our policy

Raw and processed RNA-seq, ChIP-seq, and ATAC-seq data generated in this study have been deposited to the Gene Expression Omnibus (GEO) under accession

number GSE253803. Human melanoma scRNA-seq data was obtained from GEO accession number GSE115978. The TurboID proteomics data have been deposited to the ProteomeXchange Consortium via the PRIDE partner repository with the dataset identifier PXD060265. All other relevant data supporting the key findings of this study are available within the article and its Supplementary Information files or from the corresponding authors upon request.

Research involving human participants, their data, or biological material

Policy information about studies with human participants or human data. See also policy information about sex, gender (identity/presentation), and sexual orientation and race, ethnicity and racism.

Reporting on sex and gender	No human data was generated in this study.
Reporting on race, ethnicity, or other socially relevant groupings	No human data was generated in this study.
Population characteristics	No human data was generated in this study.
Recruitment	No human data was generated in this study.
Ethics oversight	No human data was generated in this study.

Note that full information on the approval of the study protocol must also be provided in the manuscript.

Field-specific reporting

Please select the one below that is the best fit for your research. If you are not sure, read the appropriate sections before making your selection.

[x] Life sciences [ ] Behavioural & social sciences [ ] Ecological, evolutionary & environmental sciences

For a reference copy of the document with all sections, see nature.com/documents/nr-reporting-summary-flat.pdf

Life sciences study design

All studies must disclose on these points even when the disclosure is negative.

Sample size	While no formal power calculation was performed, in all cases we aimed for the sample size to be as large as possible within the technical confines of the experiment and availability of animals.
Data exclusions	No data was excluded from the analysis other than quality control filtering.
Replication	RNA-seq and ATAC-seq data has not been replicated due to technical/cost limitations, however 3 technical replicates were performed for each condition in all experiments. For all other experiments, at least n=3 biological replicates were assayed.
Randomization	No randomization was done as in all cases there was only one experimental group.
Blinding	No blinding was done as in all cases there was only one experimental group.

Reporting for specific materials, systems and methods

We require information from authors about some types of materials, experimental systems and methods used in many studies. Here, indicate whether each material, system or method listed is relevant to your study. If you are not sure if a list item applies to your research, read the appropriate section before selecting a response.

Materials & experimental systems

n/a	Involved in the study	
[ ]	[x]	Antibodies
[ ]	[x]	Eukaryotic cell lines
[x]	[ ]	Palaeontology and archaeology
[ ]	[x]	Animals and other organisms
[x]	[ ]	Clinical data
[x]	[ ]	Dual use research of concern
[x]	[ ]	Plants

Methods

n/a	Involved in the study	
[ ]	[x]	ChIP-seq
[x]	[ ]	Flow cytometry
[x]	[ ]	MRI-based neuroimaging

Antibodies

Antibodies used	Primary antibodies used were: rabbit anti-HMGB2 (abcam, ab124670 - for HMGB2 IF), rabbit anti-HMGB2 (Millipore Sigma, HPA053314 - for HMGB2 Western blot), rabbit anti-HMGB1 (abcam, ab18256), rabbit anti-HMGA1 (abcam, ab129153), mouse anti-α-

	tubulin (Millipore Sigma, CP06), chick anti-β-tubulin (Novus Biologicals, NB100-1612), mouse anti-acetylated tubulin (Millipore Sigma, 6793), rabbit anti-acetylated tubulin (Cell Signaling Technologies, CST 5335), rat anti-tyrosinated tubulin (Millipore Sigma, MAB1864-I), mouse anti-polyglutamylated tubulin (Millipore Sigma, T9822), mouse anti-GFP (abcam, ab1218), rabbit anti-H3Ac (Millipore Sigma, 06-599), mouse anti-Annexin V (Santa Cruz, sc-74438), rabbit anti-cleaved caspase-3 (CST 9661), rabbit anti-cleaved PARP (CST 5625), rabbit anti-YAP (CST 14074), mouse anti-Twist (abcam 50887), rabbit anti-Snail (CST 3879), rabbit anti-SMAD3 (abcam ab40854), rabbit anti-SYNE2 (abcam ab204308), rabbit anti-S100a6 (abcam ab204028), mouse anti-BRAF[V600E] (abcam ab228461), rabbit anti-acetylated tubulin (abcam ab179484), rabbit anti-HMGB2 (abcam ab67282 - for ChIP-seq only), rabbit anti-V5 (abcam ab9116), rabbit anti-H3K4me3 (Epicypher 13-0041). All primary antibodies were used at 1:200.
Validation	All antibodies are commonly used in our lab and have been validated by the suppliers.

Eukaryotic cell lines

Policy information about cell lines and Sex and Gender in Research

Cell line source(s)	A375, SK-MEL-5, Panc-1, MiA-PaCa-2, HTB-4, HTB-9 and HEK 293T cells were obtained from ATCC.
Authentication	A375, SK-MEL-5, Panc-1, MiA-PaCa-2, HTB-4, HTB-9 and HEK 293T cells were authenticated using Short Tandem Repeat profiling at ATCC.
Mycoplasma contamination	Cells were routinely tested to be free of mycoplasma.
Commonly misidentified lines (See ICLAC register)	None of the cell lines used in this study are commonly misidentified.

Animals and other research organisms

Policy information about studies involving animals; ARRIVE guidelines recommended for reporting animal research, and Sex and Gender in Research

Laboratory animals	Zebrafish (Danio rerio) - Genotype: casper; mitfa-BRAFV600E; p53-/-; mitfa-/-. Age: 6-12 months. Mouse (Mus musculus): athymic, 6-8 week old females.
Wild animals	No wild animals were used in the study.
Reporting on sex	Sex was not a variable in the zebrafish studies and thus was not controlled for. Female mice were used for mouse experiments.
Field-collected samples	No field-collected samples were used in the study.
Ethics oversight	All animal procedures were approved by the Memorial Sloan Kettering Cancer Center Institutional Animal Care and Use Committee (protocol #12-05-008).

Note that full information on the approval of the study protocol must also be provided in the manuscript.

Plants

Seed stocks	No plant material was used in the study.
Novel plant genotypes	No plant material was used in the study.
Authentication	No plant material was used in the study.

ChIP-seq

Data deposition

- [x] Confirm that both raw and final processed data have been deposited in a public database such as GEO.
- [x] Confirm that you have deposited or provided access to graph files (e.g. BED files) for the called peaks.

Data access links *May remain private before publication.*	The ChIP data has been added to our existing GEO repository GSE253803.
Files in database submission	FASTQ and bed files.

Genome browser session (e.g. UCSC)	No longer applicable.

Methodology

Replicates	2 replicates were performed for each experimental group and negative control.
Sequencing depth	The libraries were sequenced on an Illumina NovaSeq 6000, with ~30-40 million 100 bp paired-end reads per library.
Antibodies	anti-HMGB2: abcam 67282<br>anti-V5: abcam 9116<br>rabbit anti-H3K4me3: Epicypher 13-0041
Peak calling parameters	To ascertain enriched regions, MACS2 was used with a p-value setting of 0.001 and run against a matched control for each condition. A peak atlas was created by combining the superset of all peaks using the 'merge' function in the BEDTools suite v2.29.2. Read density profiles were created using deepTools 'bamCoverage' v3.3.0, normalized to 10 million uniquely mapped reads and with read pileups extended to 200 bp. Version 1.6.1 of featureCounts was used to build a raw counts matrix and DESeq2 was used to calculate differential enrichment for all pairwise contrasts for experiments with replicates.
Data quality	For single sample data, MACS2 was run by swapping bams of different conditions to find differential regions. Peak-gene associations were created by assigning all intragenic peaks to that gene, while intergenic peaks were assigned using linear genomic distance to transcription start site.
Software	TrimGalore (v 0.4.5), cutadapt (v 1.15), FastQC (v 0.11.5), bowtie2 (v 2.3.4.1), PicardTools (v 2.16.0), MACS2 (v 2.2.9.1), BEDTools (v 2.29.2), deepTools (v 3.3.0), featureCounts (v 1.6.1), HOMER (v 4.5).
