# Supplementary material for: Mechanical confinement governs phenotypic plasticity in melanoma
Source: Nature. 2025 Aug 27;647(8089):517–27. doi: 10.1038/s41586-025-09445-6 (PMC12611772; doi:10.1038/s41586-025-09445-6)
Supplement: Supplementary file 4 — Supplementary Tables 1–10. [file 41586_2025_9445_MOESM4_ESM.zip › 2024-01-00797D-s4/SupplementaryTablelegends.docx]

**Supplementary Table 1: Analysis of interface markers from Jerby et al. human melanoma scRNA-seq dataset.** Interface marker genes and GSEA results.

**Supplementary Table 2: RNA-seq of confined A375 cells.** Differentially expressed genes and GSEA results.

**Supplementary Table 3: Comparison of co-expressed genes between confined A375 melanoma cells and human patient interface cells.** Gene expression data and pathway analysis results.

**Supplementary Table 4: Putative HMGB2 interactor proteins identified from TurboID proximity labelling.**

**Supplementary Table 5: ATAC-seq of A375 cells overexpressing HMGB2.** Significantly enriched peaks and pathway analysis results.

**Supplementary Table 6: HMGB2 targets in A375 cells identified by ChIP-seq.** Significantly enriched peaks relative to input and HMGB2^KO^ cells.

**Supplementary Table 7: RNA-seq of A375 cells overexpressing HMGB2.** Differentially expressed genes and GSEA results.

**Supplementary Table 8: HMGB2 targets in SKMEL5 cells identified by ChIP-seq.** Significantly enriched peaks relative to input and empty vector control.

**Supplementary Table 9: RNA-seq of HMGB2^KO^ SKMEL5 cells.** Differentially expressed genes and GSEA results.

**Supplementary Table 10: Tumor volume and animal weight measurements for *in vivo* drug tolerance experiments.** Data is organized by replicate and group.
